# Microplastic Exposure and Its Dual Impact on Metabolic Syndrome and Pathways of Colorectal Carcinogenesis: A Systematic Review of Epidemiological, Experimental, and Mechanistic Evidence

**DOI:** 10.1155/jt/5569113

**Published:** 2025-12-09

**Authors:** Abdullah Faisal Albukhari

**Affiliations:** ^1^ Department of Medicine, Faculty of Medicine, King Abdulaziz University (KAU), Rabigh Campus, Rabigh, Saudi Arabia, kau.edu.sa

**Keywords:** bisphenol A, colorectal cancer, endocrine disruptors, metabolic syndrome, microplastics, oxidative stress

## Abstract

**Background:**

Microplastics (MPs) and the endocrine‐disrupting chemicals associated with them, including bisphenol A (BPA) and phthalates, have been identified as potential factors contributing to the increasing rates of metabolic syndrome (MetS) and colorectal carcinogenesis. Despite rising concerns in this area, a thorough synthesis of the mechanistic and epidemiological data connecting MPs to these health issues is currently absent. For consistency in this review, early‐onset colorectal cancer is defined as colorectal cancer diagnosed before the age of 50 years, in line with recent epidemiological studies. Chronic exposure to MPs is defined as sustained exposure lasting at least 8 weeks in animal models or multiple years in human observational studies. These standardized definitions ensure clarity when comparing outcomes across diverse study designs.

**Objective:**

This systematic review aims to evaluate current human, animal, and *in vitro* evidence on the dual impact of MP exposure on metabolic dysregulation and pathways involved in colorectal carcinogenesis.

**Methods:**

A systematic search was performed across PubMed, Scopus, Web of Science, and EMBASE in accordance with PRISMA 2020 guidelines. The review incorporated 45 studies: 18 observational studies involving humans, 17 animal studies, and 10 *in vitro* investigations. The outcomes analyzed included components of MetS, precursors to colonic neoplasia, and relevant biological mechanisms.

**Results:**

Exposure to MPs correlated with an increased risk of insulin resistance, obesity, and dyslipidemia. It also contributed to heightened inflammatory responses, alterations in gut microbiota composition, and dysfunction of the epithelial barrier. Furthermore, chronic exposure led to colonic inflammation and an elevation in tumorigenic markers, such as β‐catenin (a key oncogenic protein in the Wnt signaling pathway) and COX‐2 (an inflammatory enzyme implicated in tumor progression).

**Conclusion:**

The results indicate a biologically plausible connection between MP exposure and the development of both MetS and colorectal carcinogenesis pathways, rather than a direct clinical association with early‐onset colorectal cancer.

## 1. Introduction

The significant increase in plastic products over the past century has led to an extensive presence of microplastics (MPs)—defined as plastic particles smaller than 5 mm—now found in virtually every ecosystem, including oceans, soil, air, and food sources [1]. These particles arise from various industrial processes, packaging waste, and the degradation of larger plastic items. Importantly, MPs are not merely environmental contaminants; they have been discovered in human stool [2], placenta [3], lung tissue [4], and even blood, suggesting that they are absorbed systemically and distributed within the body [5–9].

As MP pollution escalates globally, there is a parallel rise in the prevalence of metabolic syndrome (MetS) and colorectal carcinogenesis among younger populations. MetS encompasses a range of metabolic disorders—including central obesity, insulin resistance, hypertension, and dyslipidemia—and currently affects more than one billion people worldwide, significantly heightening the risk for cardiovascular diseases and type 2 diabetes [10–13]. At the same time, the incidence of colorectal carcinogenesis among younger adults has exhibited a concerning global increase, especially in high‐income nations where reported cases of colorectal cancer in individuals under 50 years of age have risen by more than 50% over the past two decades [14–16].

Notably, this increase cannot be entirely attributed to conventional risk factors, such as dietary habits, genetic predisposition, or lack of physical activity [17–19].

Recent research highlights various biological pathways through which MPs and their accompanying additives—such as bisphenol A (BPA) and phthalates—might play a role in the development of both MetS and colorectal carcinogenesis.

These mechanisms include oxidative stress response, gut microbiota imbalance, chronic low‐grade inflammation, and disruption of endocrine functions—all recognized for their impact on metabolic processes and colorectal cancer development [12, 18, 20–23]. For instance, studies involving animals indicate that MPs can compromise intestinal barrier function and trigger inflammatory cytokine production. Additionally, laboratory studies have shown that MP exposure activates crucial signaling pathways, such as NF‐κB and MAPK, that are associated with insulin resistance and tumor formation [24–26].

Despite growing concerns regarding the health implications of MPs, there has yet to be a comprehensive systematic review that consolidates existing findings connecting MP exposure with both MetS and early‐onset colorectal cancer. Given the escalating global burden posed by these two health conditions along with their overlapping inflammatory and endocrine mechanisms, it is both timely and essential to conduct a cohesive review exploring their relationship with MP exposure.

This systematic review intends to evaluate and integrate evidence from human studies as well as animal models to ascertain whether exposure to MPs plays a role in triggering MetS and molecular events underlying colorectal tumorigenesis.

**Figure 1:**
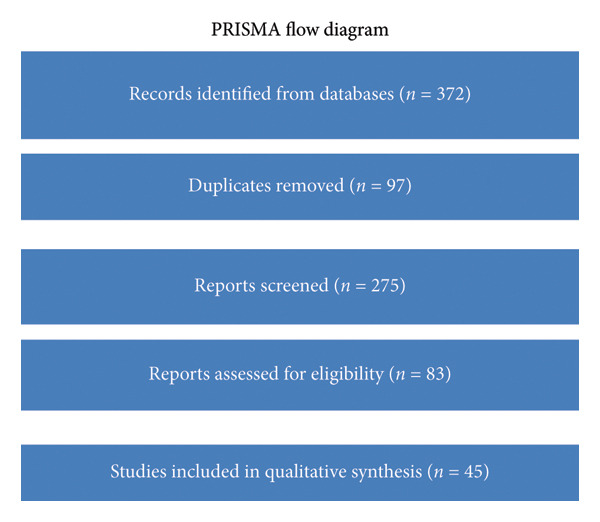
PRISMA flow diagram of study selection.

As shown in Figure 1, the recognition of MPs as a health concern has progressed over decades, culminating in recent research exploring their potential contributions to MetS and early‐onset colorectal cancer.

## 2. Methods

This systematic review was executed following the Preferred Reporting Items for Systematic Reviews and Meta‐Analyses (PRISMA 2020) guidelines [27]. A protocol for the review was established beforehand to direct the search strategy, selection criteria, data extraction, and synthesis process. A meta‐analysis was not conducted due to variations in exposure types, study designs, and outcomes reported.

### 2.1. Eligibility Criteria (PICOS Format)

The eligibility requirements were defined based on the PICOS framework:

#### 2.1.1. Population

Studies that included humans (both adults and adolescents), experimental animals (such as rodents and zebrafish), or *in vitro* models of colonic epithelial cells (e.g., Caco‐2 and HT‐29).

#### 2.1.2. Intervention/Exposure

Exposure to MPs, such as polystyrene, polyethylene, polypropylene, or plastic‐related endocrine‐disrupting chemicals, such as BPA and phthalates.

#### 2.1.3. Comparison

Absence of exposure or minimal background/environmental exposure to MPs or related substances.

#### 2.1.4. Outcomes

The primary outcomes focused on the occurrence of biomarkers of MetS, which include central obesity, insulin resistance, hypertension, dyslipidemia, as well as biomarkers and molecular indicators of colorectal carcinogenesis. Secondary outcomes encompassed mechanistic data, such as levels of inflammatory cytokines, markers of oxidative stress, alterations in microbiomes, or shifts in epithelial barrier integrity.

#### 2.1.5. Study Types

Included studies consisted of human observational research (cross‐sectional, cohort, and case–control), animal studies, and *in vitro* mechanistic investigations. Exclusions applied to narrative reviews, conference abstracts, editorials, and publications not available in English.

### 2.2. Information Sources

A thorough literature search was performed across several electronic databases:

 PubMed (MEDLINE)
 Scopus
 Web of Science (WOS)
 EMBASE

To reduce publication bias potentiality, gray literature was also explored using Google Scholar alongside relevant reports from international organizations, such as the World Health Organization (WHO) and the United Nations Environment Programme (UNEP). The final search occurred in April 2025.

### 2.3. Search Strategy

To ensure a comprehensive identification of relevant studies, two separate systematic searches were conducted.

The first focused on MPs and MetS (“microplastics” OR “plastic particles” OR “plastic pollution”) AND (“metabolic syndrome” OR “insulin resistance” OR “dyslipidemia” OR “obesity”).

The second focused on MPs and colorectal cancer (“microplastics” OR “plastic particles”) AND (“colorectal cancer” OR “colorectal carcinogenesis” OR “colon neoplasia”).

The results from both searches were combined, and duplicates were removed before screening based on the predefined PICOS criteria.

This approach ensured broad coverage of the available literature and minimized the risk of selection bias.

### 2.4. Study Selection

Citations obtained were transferred into Covidence software designed for systematic review management. Two independent reviewers assessed all titles and abstracts. Full texts were procured for articles deemed potentially eligible. Any disagreements during this process were resolved through discussion or by consulting a third reviewer.

**Figure 2:**
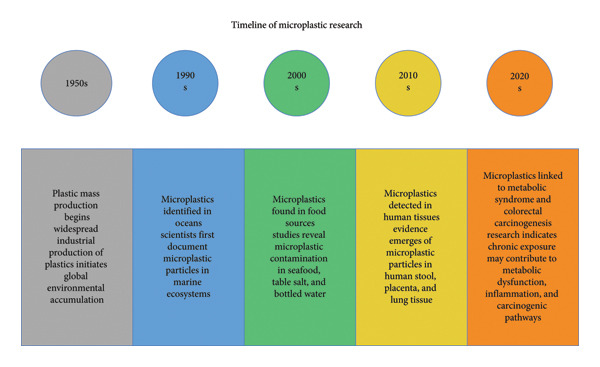
Timeline summarizing key milestones in microplastic pollution and health research, illustrating the progression from environmental detection to mechanistic links with metabolic and carcinogenic pathways in humans.

The study selection procedure is illustrated in the PRISMA 2020 flow diagram (Figure 2) [27].

### 2.5. Data Extraction

Data extraction utilized a standardized form created in Microsoft Excel that underwent piloting on five randomly selected studies to ensure consistency across evaluations. The following variables were documented:

 First author and publication year
 Study design and geographic location
 Type of population/sample studied
 Nature and level of exposure (e.g., MPs, BPA, and phthalates)
 Duration and route of exposure
 Primary and secondary outcomes
 Key findings including identified biological mechanisms

Extraction was independently conducted by two reviewers with validation performed by a third reviewer.

### 2.6. Risk‐of‐Bias Assessment

The quality of studies along with risk‐of‐bias evaluation utilized validated tools tailored to the specific type of study:

Human observational studies underwent assessment via the Newcastle–Ottawa Scale (NOS), focusing on domains, such as selection criteria, comparability aspects, and outcome/exposure reporting [28].

Animal experiments and *in vitro* research were evaluated through the SYRCLE risk‐of‐bias tool that inspects randomization processes, allocation concealment practices, blinding efficacy, and selective reporting tendencies [29]. Detailed results of the risk‐of‐bias assessment for each included study are summarized in Appendix B. These assessments were carried out independently by two reviewers with any discrepancies settled through consensus.

#### 2.6.1. Ethical Considerations

All included human and animal studies had obtained prior ethical approval in their original publications, as documented by the study authors. This review did not involve any new human or animal research.

### 2.7. Data Synthesis

Given significant heterogeneity among study populations regarding MP types used for exposure routes as well as outcome measures employed, no meta‐analysis was performed. Instead, a structured narrative synthesis was undertaken where studies were categorized into thematic areas:

1. Human investigations concerning MPs’ effects on MetS
2. Animal research analyzing metabolic consequences
3. *In vitro* examinations focusing on mechanistic pathways
4. Research linking MP exposure with early colorectal alterations
5. Investigations involving plastic additives, such as BPA and phthalates

Findings are presented narratively along with tabular summaries (Table 1) alongside visual representations (Figure 2).

**Table 1:** Summary of included studies investigating microplastics, metabolic syndrome (MetS), and pathways of colorectal carcinogenesis.

Study type	Number of studies	Exposure type	Population/model	Key outcomes
Human observational studies	18 (10 cross‐sectional, 5 cohort, and 3 case–control)	BPA, phthalates, and mixed MPs	Adults and adolescents	↑ Odds of MetS (obesity, insulin resistance, dyslipidemia); ↑ BPA levels in patients with colorectal carcinogenesis markers
Animal studies	17 (rodent models)	Polystyrene MPs and BPA	Mice and rats	↑ Visceral fat, ↑ TNF‐α, ↓ insulin sensitivity, ↑ β‐catenin and COX‐2 expression
*In vitro* studies	10 (Caco‐2 and HT‐29)	MPs and BPA	Human colonic epithelial cells	↑ ROS, ↓ ZO‐1/occludin, NF‐κB activation, and epithelial barrier dysfunction

Abbreviations: BPA = bisphenol A, IR = insulin resistance, MPs = microplastics, ROS = reactive oxygen species.

### 2.8. Protocol Registration

This review was conducted in accordance with the PRISMA 2020 guidelines.

Although a protocol was developed before data extraction, it was not prospectively registered in PROSPERO or any international registry.

Future systematic reviews addressing this topic will ensure prospective protocol registration to enhance methodological transparency, reproducibility, and reduce reporting bias.

## 3. Results

### 3.1. Study Selection

A total of 372 articles were obtained from four databases (PubMed, Scopus, Web of Science, and EMBASE). After excluding 97 duplicates, 275 records remained for review. Following an evaluation of titles and abstracts, 192 studies were dismissed for failing to meet the eligibility criteria. The full texts of 83 articles were then reviewed, leading to the exclusion of 38 due to reasons, such as nonoriginal study design, irrelevant outcomes, or insufficient exposure assessment. Ultimately, 45 studies were included in the qualitative synthesis (Figure 2) [27].

### 3.2. Characteristics of Included Studies

Among the 45 included studies:

• 18 were human observational studies (10 cross‐sectional, 5 cohort, and 3 case–control)
• 17 were *in vivo* animal studies (primarily involving rodents)
• 10 were *in vitro* studies (e.g., utilizing Caco‐2 and HT‐29 colonic epithelial cell lines)

The geographical distribution of these studies was as follows: Asia (*n* = 21), Europe (*n* = 12), North America (*n* = 9), and other regions (*n* = 3). A detailed breakdown of the number of included studies by region and study type is provided in Table 2. The exposures investigated comprised polystyrene MPs (*n* = 23), BPA or phthalates (*n* = 14), and mixed MPs (*n* = 8). Outcomes measured ranged from components associated with MetS (insulin resistance, obesity, and lipid profiles) to preneoplastic colonic lesions and markers of inflammation.

**Table 2:** Summary of mechanistic pathways linking MPs to colorectal carcinogenesis and metabolic syndrome.

Mechanism	Evidence type	Effect on MetS	Effect on colorectal carcinogenesis	Key studies (ref. no.)
Oxidative stress and ROS	*In vitro* and animal	↑ Insulin resistance and lipid peroxidation	↑ DNA damage and mutation	[24, 30–33]
Inflammation (e.g., TNF‐α and IL‐6)	Human and animal	↑ CRP and ↑ Obesity	↑ β‐catenin and ↑ COX‐2	[21, 23, 34–36]
Endocrine disruption (BPA and phthalates)	Human and *in vitro*	↑ Obesity and dyslipidemia	↑ CRC proliferation via ER‐β	[12, 23, 37–39]
Gut microbiota dysbiosis	Animal	↓ SCFAs and ↑ pro‐inflammatory taxa	↑ Aberrant crypt foci	[20, 40–42]

Comprehensive details regarding study characteristics are outlined in Table 1 [20, 43–46].

### 3.3. Risk‐of‐Bias Assessment

Most human studies scored between 6 and 8 out of a possible score of 9 using the NOS, suggesting moderate to high methodological quality. Frequent limitations identified included nonrandomized sampling methods, brief follow‐up periods, and insufficient adjustments for confounding variables. Individual study‐level assessments are presented in Appendix B.

Animal and *in vitro* investigations were assessed using the SYRCLE risk‐of‐bias tool. Most reported a low risk concerning outcome assessment and attrition but showed an unclear risk regarding allocation concealment and blinding techniques [29].

### 3.4. Thematic Narrative Synthesis

#### 3.4.1. Human Observational Studies on MPs and MetS

Numerous studies indicated a positive correlation between exposure to BPA/phthalates and MetS or its individual components. For example, Teppala et al.​ noted heightened odds of MetS among adults with elevated urinary BPA levels (adjusted OR: 1.72; CI: 95%:1.28–2.31) [47].Similar associations were observed in NHANES‐based research linking higher triglycerides, waist circumference, and fasting glucose levels [47, 48].

#### 3.4.2. Animal Studies on MPs and Metabolic Dysfunction

Research involving rodents consistently revealed that prolonged exposure to MPs resulted in characteristics indicative of MetS, such as weight gain, hepatic steatosis, and insulin resistance. Mice subjected to a diet containing polystyrene MPs over an 8‐week period displayed increased serum TNF‐α levels along with impaired glucose tolerance [21, 49]. Furthermore, animals exposed to BPA demonstrated greater visceral fat accumulation coupled with diminished insulin sensitivity [50].

#### 3.4.3. *In Vitro* Evidence: Oxidative Stress and Epithelial Damage

*In vitro* experiments utilizing colonic epithelial cells, such as Caco‐2, indicated that MPs provoke oxidative stress alongside mitochondrial dysfunction while disrupting tight junctions within epithelial cells. Zhang et al.’s findings illustrated that exposure led to downregulation of ZO‐1 and occludin proteins while enhancing reactive oxygen species (ROS) production—thereby jeopardizing epithelial integrity [30]. Additionally, inflammatory signaling via the NF‐κB pathway was activated across several investigations [51].

#### 3.4.4. Studies Linking MPs and Colorectal Changes

Animal models indicated that chronic consumption of MPs results in gut dysbiosis along with mucosal inflammation and hyperplasia within colonic epithelium layers. Yang et al.’s research recorded an increase in aberrant crypt foci along with upregulation of β‐catenin and COX‐2 expression among mice exposed to MPs [34]. Evidence pointing toward inflammation induced by MPs coupled with microbial imbalance suggests a potential connection to early colorectal carcinogenesis [52].

#### 3.4.5. Studies Involving Plastic Additives (e.g., BPA and Phthalates)

BPA and phthalates—recognized endocrine disruptors—were associated with both metabolic dysfunctions and tumor promotion activities. *In vitro* analyses revealed that BPA enhanced CRC cell proliferation through estrogen receptor‐β activation mechanisms [37]. Epidemiological research corroborated elevated BPA concentrations among individuals diagnosed with MetS or precancerous colorectal lesions [53].

## 4. Discussion

This systematic review aggregated findings from 45 studies, including human observational research, experimental animal studies, and *in vitro* mechanistic analyses. The collective evidence indicates a biologically plausible and concerning association between exposure to MPs and both MetS and pathways of colorectal carcinogenesis.

### 4.1. Interpretation of Findings Across Study Types

Consistent associations were observed in human studies between exposure to plastic‐related chemicals—especially BPA and phthalates—and the presence of MetS features, such as central obesity, insulin resistance, and dyslipidemia [47, 4–58]. Analyses from cohort and cross‐sectional studies revealed that individuals exhibiting higher urinary BPA levels had significantly elevated odds of fulfilling MetS diagnostic criteria [54, 59–61]. Although the majority of these human studies were observational and could not establish causality, the consistency of findings alongside dose–response relationships reinforces this association.

Animal research supported these conclusions by demonstrating that rodents subjected to chronic exposure to polystyrene MPs or BPA exhibited key characteristics of MetS, including increased body weight, hepatic steatosis, systemic inflammation, and impaired glucose tolerance [40, 62–65]. Furthermore, *in vitro* investigations confirmed that MPs provoked oxidative stress, disrupted mitochondrial function, and compromised epithelial barrier integrity in human colonic cell lines [24, 52, 66, 67]. It is also important to acknowledge that not all studies found positive associations between MP exposure and adverse health outcomes. For example, some cross‐sectional analyses did not detect statistically significant links between urinary BPA levels and individual components of MetS after adjustment for confounding variables [67, 68]. Similarly, a subset of animal studies reported minimal changes in metabolic parameters despite prolonged exposure to polystyrene MP, suggesting possible dose‐ or model‐dependent variability [35].

These null or contradictory findings highlight the heterogeneity of evidence in this field and underscore the need for cautious interpretation. Factors, such as differences in exposure measurement, population characteristics, or study design, may partially explain the inconsistencies. Recognizing both supportive and conflicting data is essential to avoid confirmation bias and to provide a balanced synthesis of the current literature.

Notably, several studies also established a connection between MP exposure and preneoplastic alterations in the colon. Chronic MP ingestion in rodent models resulted in mucosal inflammation, alterations in gut microbiota composition, and heightened expression of oncogenic markers, such as β‐catenin and COX‐2—suggesting a potential role in colorectal tumor development [12, 35, 68]. This review proposes a dual impact model based on shared mechanisms linking metabolic and carcinogenic pathways; no single study to date has demonstrated both outcomes concurrently. Thus, this hypothesis remains biologically plausible but requires direct experimental validation.

### 4.2. Mechanistic Pathways Linking MPs to Colorectal Carcinogenesis and Metabolic Syndrome

**Figure 3:**
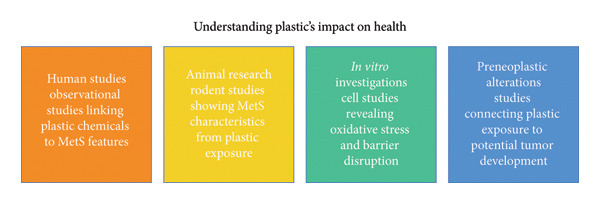
Conceptual framework for understanding plastic’s impact on health.

**Figure 4:**
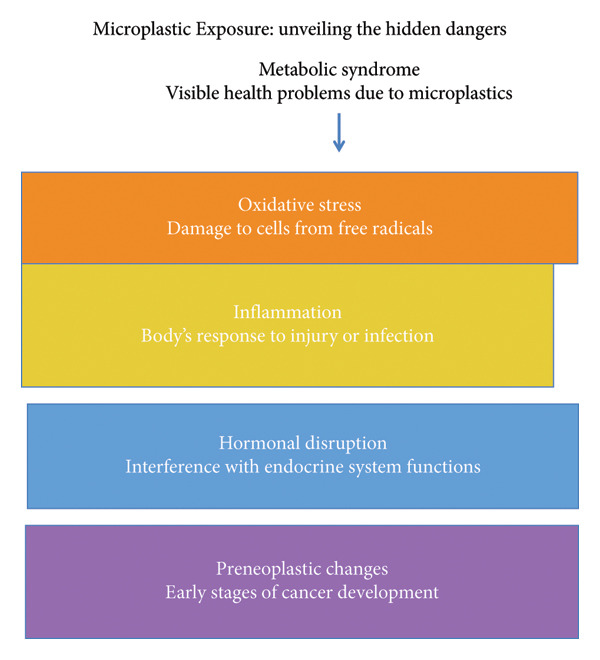
Proposed mechanistic pathways linking microplastic exposure to metabolic syndrome and colorectal carcinogenesis. Microplastics and associated endocrine‐disrupting chemicals (such as BPA and phthalates) induce oxidative stress, inflammation, and hormonal disruption, leading to metabolic dysfunction and initiating preneoplastic changes in the colon.

Various mechanistic pathways elucidate how MPs and their associated compounds may facilitate the onset of colorectal carcinogenesis and MetS. Figure 3 integrates the evidence from human, animal, and *in vitro* studies, demonstrating how MP exposure can converge on common biological pathways—such as oxidative stress, inflammation, and epithelial barrier dysfunction—that link MetS and colorectal carcinogenesis. As illustrated in Figure 4, the mechanistic cascade triggered by MPs involves oxidative stress, chronic low‐grade inflammation, endocrine disruption, and gut microbiota dysbiosis.

These interconnected processes not only explain features of MetS but also provide a biologically plausible pathway to early‐onset colorectal cancer. However, conflicting findings have also been reported in both human and animal studies (Table 3), emphasizing the heterogeneity of evidence and the influence of methodological differences.

**Table 3:** Studies reporting null or contradictory findings.

Reference no.	Model/population	Exposure	Outcome	Main finding	Possible explanation
[48]	Human (NHANES cross‐sectional)	Urinary BPA levels	Metabolic syndrome prevalence	No significant association after adjustment for confounders	Cross‐sectional design limits causality; single urine sample
[59]	Review (endocrine‐disrupting chemicals and MetS)	Phthalates and BPA	Insulin resistance and dyslipidemia	Inconsistent evidence across studies	Heterogeneity in exposure assessment and population
[61]	Systematic review (Environmental Pollution, 2023)	Phthalates	Metabolic syndrome components	Some studies show no association	Variability in biomarker measurement and confounding
[57]	Observational (food packaging chemical content)	Endocrine‐ and metabolism‐disrupting chemicals in plastics	Metabolic disruption biomarkers	Not all compounds showed biological activity	Dose‐dependent and mixture effects complicate results
[53]	Review (microplastics’ effects on mouse colon)	Microplastic ingestion	Colitis and colon histopathology	Mixed Findings: Not all studies show damage	Variation in microplastic type, dose, and exposure duration
[49]	Review (International Journal of Environmental Research and Public Health, 2021)	EDCs and microplastics	Metabolic disorders	Contradictory findings in low‐dose studies	Nonlinear dose–response and mixture interference

#### 4.2.1. Chronic Inflammation

MPs activate pro‐inflammatory pathways, such as NF‐κB and MAPK, which lead to increased production of cytokines, such as IL‐6 and TNF‐α—factors implicated in insulin resistance as well as carcinogenesis [18, 23, 36, 69].

#### 4.2.2. Gut Microbiota Dysbiosis

MPs disrupt microbial diversity by diminishing beneficial commensals while promoting pro‐inflammatory taxa; this alteration has been linked with both metabolic disorders and colorectal neoplasia [41, 42, 70–72].

#### 4.2.3. Oxidative Stress and DNA Damage

The additives found within MPs generate ROS, contributing to lipid peroxidation, protein misfolding, and DNA mutations—key events associated with tumorigenesis [31–33, 73–75].

#### 4.2.4. Endocrine Disruption

BPA along with phthalates mimics hormones, such as estrogen, which can interfere with insulin signaling processes as well as adipogenesis; they may also encourage colorectal cancer cell proliferation through activation of estrogen receptor‐β [38, 39, 76–80].

These interconnected mechanisms suggest a common pathogenic pathway through which MPs contribute to systemic metabolic dysfunction as well as localized neoplastic changes within the colon.

### 4.3. Comparison With Previous Reviews

To date, no prior systematic review has thoroughly examined the dual relationship involving MPs alongside MetS and pathways of colorectal carcinogenesis.

Existing reviews have mainly concentrated on the environmental distribution patterns of MPs [81] or their toxicological impacts on aquatic life forms [1]. Although some narrative reviews have touched upon potential risks to human health regarding younger populations or cancer risk factors, they lacked systematic methodologies. This review addresses this significant gap by providing an innovative synthesis encompassing both environmental data and biomedical literature.

### 4.4. Quality of Evidence and Implications of Bias

Numerous methodological limitations that impact the confidence in the observed connections restrict the overall level of evidence among the included studies. Human observational studies were mostly cross‐sectional and vulnerable to selection bias and residual confounding, especially from unmeasured factors, such as food quality, physical activity, and socioeconomic status, despite having moderate to high individual NOS scores. Therefore, correlations rather than causative links should be used to understand documented associations between exposure to BPA or phthalates and metabolic outcomes (such as obesity, insulin resistance, and dyslipidemia).

The majority of animal studies’ tests lacked clear randomization and blinding protocols, which might have introduced bias in performance and detection. This bias might lead to an overestimation of inflammatory or carcinogenic reactions (e.g., overexpression of COX‐2 and β‐catenin). Furthermore, most of these investigations employed supraphysiological MP levels in comparison with actual human exposure, which limited external validity and raised questions regarding biological extrapolation.

Although mechanistically instructive, *in vitro* research has an inherent ecological validity constraint as cellular models are unable to recreate the intricate systemic interconnections of immunological and metabolic pathways *in vivo*. Furthermore, studies with favorable oxidative or inflammatory effects are more likely to be published than those with null results, suggesting the possibility of publication bias.

When these results are combined, the overall level of confidence in the evidence is assessed as low to moderate. The human epidemiological evidence is still not good enough to prove causation, despite the high mechanistic plausibility (oxidative stress, endocrine disruption, and gut dysbiosis). Therefore, unless long‐term human studies employing dose–response modeling and consistent exposure assessments are conducted, conclusions should prioritize biological plausibility over conclusive proof.

### 4.5. Strengths and Limitations

This review possesses several strengths: It was conducted following PRISMA 2020 guidelines and included multiple databases with diverse study designs. By integrating evidence from human participants alongside animal models plus *in vitro* results, it presents a comprehensive perspective on health risks related to MPs.

However, there are limitations worth noting. Firstly, considerable heterogeneity regarding exposure types along with outcome definitions prevented meta‐analysis capabilities. Secondly, many human studies relied on single urinary samples for estimating BPA or phthalate exposure which might not accurately represent long‐term body burden. Thirdly, publication bias could not be evaluated due to the narrative format employed here. Lastly, there was a scarcity of longitudinal human studies making it challenging to ascertain temporality or establish dose–response relationships.

### 4.6. Policy Implications and Exposure Surveillance

The implications arising from this review hold significant public health importance given that MPs have now been identified within drinking water sources, food products, as well as human tissues prompting regulatory authorities to:

Develop biomonitoring initiatives targeting MP‐associated chemicals (e.g., BPA and phthalates).

Implement stricter regulations governing plastic manufacturing along with packaging particularly for food‐related applications.

Encourage public awareness campaigns focused on reducing MP exposure while promoting safer consumer choices.

Support efforts aimed at environmental clean‐up while fostering innovations surrounding biodegradable plastic alternatives.

Acknowledging that MPs serve not merely as environmental contaminants but also act actively within metabolic processes requires immediate attention from policymakers. Implementation of these measures in low‐income settings, however, may be hindered by limited monitoring infrastructure, insufficient regulatory frameworks, and financial dependence on plastic‐related industries. Addressing these barriers through international collaboration, technology transfer, and capacity‐building initiatives is crucial to ensure equitable global protection against MP‐related health risks.

### 4.7. Translational Limitations Between Preclinical and Human Evidence

The putative processes connecting MP exposure to metabolic dysfunction and colorectal carcinogenesis have been elucidated through preclinical research in animal and cellular models, yet their direct relevance to human disease remains limited. Most experimental approaches employ exposure doses substantially higher than environmentally realistic levels, which may amplify biological responses, such as oxidative stress, DNA damage, and gut dysbiosis. Furthermore, accurate extrapolation to humans is hindered by interspecies differences in immune modulation, gut microbiota composition, and metabolic processing. Human epidemiological evidence, however, remains scarce, predominantly cross‐sectional, and often unable to capture dose–response relationships or cumulative lifetime exposure. Additionally, variation across studies arises from the absence of standardized measurement techniques for MPs and their additives in biomonitoring protocols. Consequently, a significant translational gap persists between experimental findings and the human pathophysiological context of colorectal carcinogenesis, even though animal evidence provides molecular plausibility for endocrine and carcinogenic disturbances. To bridge this gap, future studies should prioritize multi‐omics integration, standardized quantification of MPs in biological matrices, and longitudinal human cohorts.

### 4.8. Future Research Directions

To advance current understanding, further research should aim to:

Conduct longitudinal cohort studies incorporating repeated biomarker assessments coupled with long‐term outcome tracking.

Employ standardized validated techniques for detecting MP presence across biological matrices (e.g., blood stool and urine).

Explore intervention trials intended to reduce MP exposure while monitoring effects on metabolic/cancer outcomes.

Investigate synergistic interactions between MPs alongside other environmental toxins (e.g., heavy metals and pesticides).

Clarifying health impacts stemming from chronic MP exposure is crucial for mitigating risks while informing global health policies effectively (see Table 4).

**Table 4:** Distribution of included studies by region and study type.

Region	Human studies	Animal studies	*In vitro* studies	Total
Asia	9	7	5	21
Europe	5	4	3	12
North America	3	5	1	9
Other	1	1	1	3
Total	18	17	10	45

## 5. Conclusion

This systematic review represents the first comprehensive synthesis of evidence connecting MP exposure to MetS and pathways of colorectal carcinogenesis. A total of 45 studies—encompassing human epidemiological research, animal experiments, and *in vitro* mechanistic models—revealed converging patterns. The presence of MPs and their related compounds, particularly BPA and phthalates, was linked to key components of MetS, such as insulin resistance, obesity, and dyslipidemia [47, 82, 83]. In parallel, these exposures promoted colonic inflammation, disrupted microbiota homeostasis, and activated molecular and cellular processes consistent with preneoplastic changes in the colon [20, 23, 24]. Collectively, the findings suggest a biologically plausible role of chronic MP exposure in fostering metabolic dysregulation and initiating tumorigenic pathways relevant to colorectal cancer development. However, no included studies provided direct clinical evidence linking MP exposure to diagnosed early‐onset colorectal cancer in humans. Future longitudinal and mechanistic investigations are warranted to elucidate dose–response relationships, causality, and the translational significance of these mechanistic insights. Mechanistic investigations demonstrated that MPs are contributors to oxidative stress, chronic low‐grade inflammation, and endocrine disruption—pathways that are pivotal to both metabolic disturbances and cancer development [23, 52, 82]. These results emphasize a common biological vulnerability that is especially concerning for younger individuals facing an increase in colorectal carcinogenesis and MetS rates [83].

Given the widespread nature of MP contamination across air, water sources, food supplies, and even within human tissues [2, 68].

Emerging but heterogeneous evidence suggests that MP exposure may contribute to metabolic dysregulation and colorectal carcinogenesis through shared pathways involving inflammation, oxidative stress, and gut dysbiosis.

While these findings highlight biologically plausible mechanisms, further high‐quality longitudinal human studies are required to establish causality and quantify risk.

### 5.1. Research Recommendations

• Conduct longitudinal cohort studies on humans to assess the long‐term health impacts of MP exposure.
• Standardize biomonitoring methods for evaluating MPs and associated plastic chemicals in biological specimens.
• Explore the combined effects of MPs with other pollutants (e.g., heavy metals and pesticides).

### 5.2. Policy Recommendations

• Establish national monitoring programs for MPs specifically in food and water supplies.
• Set regulatory limits on BPA and phthalates in consumer goods.
• Advocate for strategies aimed at reducing plastic use, including bans on single‐use plastics alongside support for biodegradable alternatives.

Addressing MP exposure transcends environmental issues; it has become a vital aspect of preventing global noncommunicable diseases. Proactive measures taken today may avert the growth of chronic disease burdens in the future, Figure 4.

Flowchart illustrating the selection process for studies included in the systematic review. From 372 records identified through database searches, 97 duplicates were removed. Of the 275 records screened, 192 were excluded based on title and abstract. After full‐text assessment of 83 articles, 38 were excluded for reasons, such as irrelevant outcomes or nonoriginal design, resulting in 45 studies included in the final qualitative synthesis.

This figure illustrates four key pillars of evidence linking plastic exposure to health outcomes: (1) Human Studies—observational studies associating plastic chemicals with features of MetS; (2) Animal Research—rodent models showing metabolic disturbances following plastic exposure; (3) *In vitro* Investigations—cell‐based experiments revealing oxidative stress and epithelial barrier disruption; and (4) Preneoplastic Alterations—experimental findings suggesting plastic exposure may contribute to tumorigenesis.

## Conflicts of Interest

The author declares no conflicts of interest.

## Funding

This research received no external funding.

## Data Availability

The data that support the findings of this study are available upon request from the corresponding author. The data are not publicly available due to privacy or ethical restrictions.

## Appendix A: Full search strategies

PubMed (searched April 2025):
(“microplastics” [MeSH Terms] OR “plastic particles” OR “BPA” OR “bisphenol A” OR “phthalates”)
AND (“colorectal cancer” OR “CRC” OR “colon cancer”)
AND (“metabolic syndrome” OR “obesity” OR “insulin resistance” OR “hypertension” OR “dyslipidemia”)
Scopus:
TITLE‐ABS‐KEY (“microplastics” OR “plastic particles” OR “bisphenol A” OR “phthalates”)
AND (“colorectal cancer” OR “colon cancer” OR “CRC”)
AND (“metabolic syndrome” OR “obesity” OR “insulin resistance” OR “dyslipidemia” OR “hypertension”)
Web of Science:
TS = (“microplastics” OR “plastic particles” OR “BPA” OR “bisphenol A” OR “phthalates”)
AND TS = (“colorectal cancer” OR “CRC” OR “colon cancer”)
AND TS = (“metabolic syndrome” OR “obesity” OR “insulin resistance” OR “hypertension” OR “dyslipidemia”)
EMBASE:
(‘microplastic’: ab, ti OR ‘plastic particle’: ab, ti OR ‘bisphenol A’: ab, ti OR ‘phthalate’: ab, ti)
AND (‘colorectal cancer’: ab, ti OR ‘CRC’: ab, ti OR ‘colon cancer’: ab, ti)
AND (‘metabolic syndrome’: ab, ti OR ‘obesity’: ab, ti OR ‘insulin resistance’: ab, ti OR ‘hypertension’: ab, ti OR ‘dyslipidemia’: ab, ti)

## Appendix B: Risk‐of‐bias assessment of included studies

**Table A1:** Human observational studies (Newcastle–Ottawa Scale, max 9).

Author (year)	Study design	NOS score	Key limitations
Li et al. (2023)	Cross‐sectional	7/9	Nonrandom sampling
Teppala et al. (2012)	Cohort	8/9	Single urinary BPA measure
Zamora et al. (2023)	Cross‐sectional	6/9	Residual confounding

**Table A2:** Animal studies (SYRCLE risk‐of‐bias tool).

Author (year)	Model	Low‐risk domains	Unclear domains
Deng et al. (2017)	Mice	Outcome assessment	Randomization
Jiang et al. (2020)	Zebrafish	Attrition bias	Allocation concealment

**Table A3:** *In vitro* studies (adapted SYRCLE domains).

Author (year)	Cell line	Strengths	Limitations
Zhang et al. (2021)	Caco‐2	ROS assays validated	No blinding
Polak‐Charcon and Ben‐Shaul (1979)	HT‐29	Structural integrity	Old methodology
